# In Vitro Model of Vascular Remodeling Under Microfluidic Perfusion

**DOI:** 10.3390/mi16010014

**Published:** 2024-12-26

**Authors:** Kotaro Nishikata, Kimisato Doi, Nobuyoshi Kaneoya, Masataka Nakamura, Nobuyuki Futai

**Affiliations:** Department of Mechanical Engineering, Shibaura Institute of Technology, 3-7-5 Toyosu, Koto-ku, Tokyo 135-8548, Japan; nb22110@shibaura-it.ac.jp (K.N.); aa20055@shibaura-it.ac.jp (K.D.); md23038@shibaura-it.ac.jp (N.K.);

**Keywords:** remodeling, spontaneous, vascular network, microfluidic, centrality

## Abstract

We developed a portable microfluidic system that combines spontaneous lumen formation from human umbilical endothelial cells (HUVECs) in fibrin–collagen hydrogels with active perfusion controlled by a braille actuator. Adaptive interstitial flow and feedthrough perfusion switching enabled the successful culture of spontaneously formed naturally branched lumens for more than one month. We obtained many large-area (2 mm × 3 mm) long-term (more than 30 days per run) time-lapse image datasets of the in vitro luminal network using this microfluidic system. We also developed an automatic image analysis pipeline to extract the morphology of the lumen network and node–edge network structure weighted with segmentwise flow parameters. The automatic lumen area measurements revealed that almost all lumens were successfully cultured in this system for approximately 50 days, following the meshwork, sprouting, remodeling, stability, and erosion stages. We found that the optimization of the lumen network during the remodeling stage can be explained by the decrease in the betweenness centrality of the WSS-weighted network and the increase in the strength centrality of the flow-rate-weighted network.

## 1. Introduction

An isotropic mesh of narrow blood vessels becomes a well-structured, high-flow vessel network. The concept of such vascular remodeling is that endothelial cells stimulated with flow shear stress and mechanical load on the cells themselves reshape in various ways, such as pruning [1], angioadaption [2], coalescent angiogenesis [3], and intussusceptive [4] angiogenesis, toward an optimal distribution of blood vessels. Culturing vascular networks in vitro will lead to a better understanding of this formation process and address the ethical issues and limitations [5,6,7] of in vivo culture using various animal models [8,9,10,11,12,13].

Morphological changes such as pruning, angioadaption, and coalescent angiogenesis do not appear quickly, so long-term culture is desirable for observation. In the vascularization of organs, co-culture of the vascular network is essential for maintaining the long-term survival and function of thick, three-dimensional tissues [14]. By focusing on the process of blood vessel formation, understanding the effects of blood flow on endothelial cells and the microvascular network, and maintaining this over time, you will be able to build a hierarchical vasculature that is closer to the body, leading to accurate modeling and drug screening in co-culture systems with larger cancer models that include functional blood vessels.

Microfluidic platforms have been used in vitro to study the effects of flow and wall shear stress on endothelial cells and microvascular networks, which can be broadly divided into pre-designed and self-organized methods. Pre-design methods, such as 3D bioprinting [15], PDMS rods [16], and needles, which use sacrificial materials to create artificial blood vessels, contribute to the stability and reproducibility of culture, although it is difficult to reproduce the physiological morphology and developmental processes of natural blood vessels, and the size of the lumen that can be formed is also limited. In contrast, self-assembled vascular networks have the potential to construct natural microvascular networks in vitro that are similar to the angiogenesis and vasculogenesis observed in vivo [17,18,19,20,21,22,23,24]. Most of these are cultured in hydrogels introduced into microchannels divided by pillar arrays or phase guides or by introducing cells into adjacent microchannels to promote germination into the hydrogel and perfusion culture. This latter approach promotes the self-organization of lumens within the hydrogel. A more straightforward system has a portable hydrostatic culture system comprising a dish with partitions [25]. Additionally, there are other examples of research that combine pre-designed methods with self-organization [22] and methods that employ patterning using sound and surface acoustic waves [20].

For the in silico approach, the synthesis of the vascular network and analysis based on principles that drive remodeling were also explored, such as material cost optimization of the whole network [26], power cost optimization, Murray’s law with SALVO global optimization [27], maximized spatial uniformity of flow rates [28], and the minimum total energy principle [29]. This synthesized network was used as a metric for real vascular networks by comparing artificial networks with image data [30].

However, the microfluidic systems for on-chip angiogenesis or vasculogenesis have yet to realize the long-term maintenance of microvascular networks. Although remodeling requires a month-long culture, few cases in which the culture time is longer than three weeks have been reported. As previously stated, incorporating the pre-designed types contributes to the stability and reproducibility of the culture. However, it is challenging to reproduce the physiological state and developmental process of the actual vascular tissue, and the size of the lumen that can be formed is also limited. It can be argued that the acoustic patterning method is partially self-organizing; however, the validity of the patterned morphology remains questionable. Most in vitro vascular network culture perfusion systems employ hydrostatic pressure or syringe pumps. However, these systems are constrained by their inability to circulate the flow, maintain a constant flow rate, and accommodate complex external piping. Furthermore, the low flow rate of the hydrostatic pressure drive impedes the diffusion of substances into the culture chamber. Consequently, an active micropump integrated into the cell culture module is a superior option for long-term culture. In addition, the effectiveness of analyzing vascular remodeling can be limited by the capabilities of visualization and automated image analysis techniques [31]. Conventional visualization of spontaneous lumen formation in vitro is often performed at coarse periods (e.g., every few days). Some morphology analyses at finer time intervals are available but limited to short periods (e.g., a few days).

Most in vitro lumen morphology analyses are limited to evaluating simple parameters (lumen diameter, vessel area, branching, and length) [18,21,23]. In some cases, computational fluid dynamics (CFD) analysis of the lumen morphology is performed [21,22]. Nevertheless, it is limited to discussing what can be read from the velocity field contour plots or the temporal changes in mean flow velocity or wall shear stress (WSS). In other words, luminal morphology cannot be quantified from flow and topological perspectives. Conversely, synthetic lumens mimic the morphological parameters (diameter, area, branch number, and segment length) and the self-similarity of commonly used in vivo angiogenesis models. Therefore, whether they can be applied to assess the functionality of spontaneous in vitro vascular models is still being determined.

We developed a portable microfluidic system that combines spontaneous lumen formation from human umbilical endothelial cells (HUVECs) in fibrin–collagen hydrogels with active perfusion controlled by a braille actuator. This system allows time-lapse observation under a microscope with a glass heater attached, and since the strength of the interstitial flow and the direction of perfusion can be easily changed, perfusion can be performed according to the stage of lumen formation. The above microfluidic system was used to create a long-distance (2 mm) lumen network in vitro, followed by a long-term time-lapse recording of angiogenesis and subsequent remodeling processes over one month. The lumen hydrodynamic parameters (flow and WSS) and the node–edge network structure were extracted from the time-lapse images. The above hydrodynamic parameters were assigned to each edge of the network to form a weighted network. The weighted network structure was then used to evaluate the optimization index during remodeling to determine whether the entire lumen network was optimized.

## 2. Experimental

### 2.1. Fabrication of the Microfluidic Device

The microfluidic device for long-term perfusion cell culture has a poly (dimethylsiloxane) (PDMS)-made microchannel covered with a glass substrate and poly (methylmethacrylate) (PMMA) reservoir. A PDMS reservoir bonded to the PMMA reservoir separates the inside into two regions: cell culture media and a bicarbonate buffer for on-chip incubation. The bicarbonate–carbonate buffer in the CO_2_ reservoir maintained a pCO_2_ level near 5% and osmolality within a physiological range. The configuration of this microfluidic device is almost the same as that reported previously [32]. However, the PDMS reservoir’s design, the microchannel’s pillar design, and the Braille cell product and pumping scheme are different.

The microchannel comprised a microchannel-patterned layer and membrane layer bonded to form a closed channel and cell culture chamber. A part of the microchannel was outside the PMMA reservoir, and a braille actuator fixture was placed on it.

**Figure 1:**
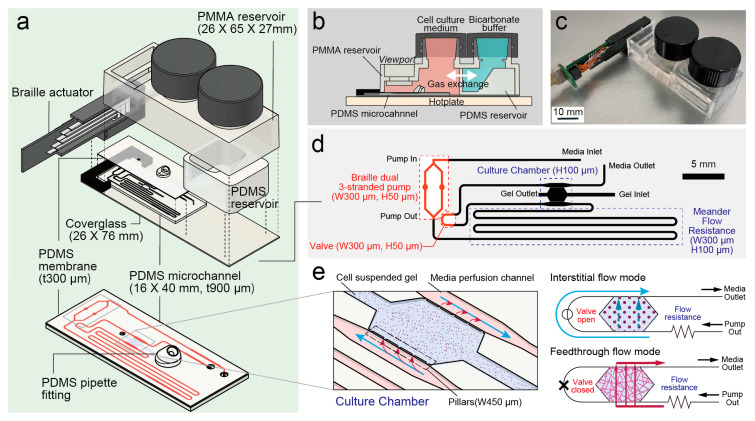
Integrated microfluidic device for perfusion culture of the microvascular network. (**a**) Construction of the microfluidic device. (**b**) An on-chip incubation system using a dual reservoir is used. (**c**) Appearance of a device with a braille cell attached. (**d**) Channel layout. A pulsation-free pumping scheme with dual 3-strand peristaltic micropumps is described. (**e**) Cell culture chamber and two perfusion modes: interstitial and feedthrough.

A microchannel-patterned layer was fabricated using a soft lithography process. A master mold was fabricated using backside photolithography [33], which involves patterning negative photoresist (SU-8 3050, Kayaku Advanced Materials, Tokyo, Japan) on a coverglass (C050701, Matsunami, Tokyo, Japan). A 50-μm photoresist layer was exposed to diffuse UV radiation (365 nm, 10 mJ/cm^2^) from the backside to form the braille pump and valve regions (see Figure 1d). After development, a 100-μm photoresist layer was coated again. All other areas were patterned by conventional collimated UV (365 nm, 10 mJ/cm^2^). Positive replicas with channel features were fabricated by molding PDMS (KE-106, Shin-Etsu Chemical, Tokyo, Japan) against the master mold. The PDMS base with a curing agent at 10:1 (*w*/*w*) was poured on the master mold and cured in an oven at 65 °C for 3 h and 120 °C for 10 min.

A 300 μm-thick PDMS membrane was formed by spin coating (350 rpm, 30 s) a PDMS prepolymer onto a glass plate and curing as described above. Both PDMS layers were treated by vacuum air plasma (20 Pa, 1.0 mA for 30 s) in a plasma coater (SC-708, Sanyu Electron, Tokyo, Japan) and assembled by direct bonding. The gel inlet and outlet were punched from the assembled PDMS microchannel using a 1 mm biopsy punch; the media inlet and outlet used a 1.5 mm biopsy punch. A PDMS microchannel and a 0.5 mm-thick glass slide (26 × 76 mm No. 5, Matsunami) were then plasma bonded as described above with the membrane layer on top.

To facilitate gel injection into the microfluidic channel, a PDMS fitting fabricated from a plastic master mold was plasma bonded to the gel inlet of the microchannel. PDMS reservoirs were also cast from plastic molds. Braille actuator fixtures and a master mold of a PDMS fitting and reservoir were 3D printed (Ultimaker 3, Ultimaker, Geldermalsen, The Netherlands).

Finally, a channel-glass assembly, a PMMA reservoir (Proto Labs, Maple Plain, MN, USA), and a PDMS reservoir were assembled using silicone adhesive (KE-42, Shin-Etsu Chemical). A braille actuator fixture was attached to the glass assembly using epoxy adhesive (Bond E-set, Konishi, Osaka, Japan).

### 2.2. Cell Culture

The human umbilical vein endothelial cells (HUVECs, C2517A, Lonza, Basel, Switzerland) were maintained in EGM-2 (Lonza), and cells at passages 5 to 6 were used for the experiments. Human lung fibroblasts (hLFs) (CC-2512, Lonza) were maintained in FGM-2 (Lonza), and cells at passages 5 to 7 were used. Both cell lines were maintained at 37 °C and 5% CO_2_ in an incubator.

When the hLFs had grown and reached subconfluency in a T-25 flask, the medium was replaced with EGM-2, and the cells were incubated with CO_2_ overnight. The medium was then collected, and 0.2-μm-thick layers of cellular debris were removed. A 1:40 mixture of hLF-conditioned and fresh EGM-2 was used for microvascular culture.

### 2.3. Construction of the Microvascular Network

A matrix solution was prepared by dissolving 10 mg/mL (final) fibrinogen (F8630, Sigma-Aldrich, Burlington, MA, USA) and 0.2 mg/mL type I collagen (354236, Corning, Corning, NY, USA) in HEPES-buffered saline solution (HBSS, C-40010, PromoCell, Heidelberg, Germany). Subconfluent HUVECs were detached using Accutase (Innovative Cell Technologies, San Diego, CA, USA), suspended in HBSS, and pelleted by centrifugation. The HUVECs were resuspended in the matrix solution to a final cell density of 8.0 × 10^6^ cells/mL. The suspension was mixed with thrombin from bovine plasma (T4648, Sigma-Aldrich) to a final concentration of 0.2 U/mL and quickly loaded into the microchamber (area: 3 mm × 2 mm, height: 100 μm) Figure 1d,e through a pipette tip fitting.

After allowing gel polymerization in a CO_2_ incubator for 5 min, the entire microchannel was primed with hLF-conditioned EGM-2. The PMMA reservoir was filled with 4 mL of the medium, and the PDMS reservoir was filled with 1.5 mL of bicarbonate buffer (0.8 M NaHCO_3_, 65 mM Na_2_CO_3_), as shown in Figure 1b.

A braille cell (SC9, KGS, Saitama, Japan) was attached to the microfluidic device, and the cell culture medium circulated in the microchannel under the peristaltic actions of braille pins. A custom USB bus-powered microcontroller-controlled piezoelectric amplifier actuated the pins of the braille cell following a constant-flow rate waveform [34]. The period of the waveform was 4 [s]. To reduce pulsation caused by blank periods in peristaltic actions, we used dual 3-strand pumps (see Figure 1d) driven half a cycle apart. The medium was circulated initially using the interstitial flow mode, as shown in Figure 1e. The media and bicarbonate buffers were replaced every three days. After the route of the lumens connecting the two side channels was confirmed, the flow mode was switched to feedthrough flow mode by closing a valve using a braille pin. The direction of the feedthrough flow was changed every 24 h.

The device was placed on a 37 °C hotplate. The medium and bicarbonate buffer were exchanged every three days. The HUVECs in the microfluidic chamber were imaged via phase-contrast microscopy (for days 0~5) and brightfield microscopy (after day 5) using an inverted microscope (DMi8 or DMIL LED, Leica, Wetzlar, Germany) with a CMOS camera (DMK33UX174, The Imaging Source, Bremen, Germany). The images were 1920 × 1200-pixel grayscale and taken every 1 h to 3 days, typically every 1 d, depending on the availability of the microscope.

### 2.4. Image Acquisition and Processing

**Figure 2:**
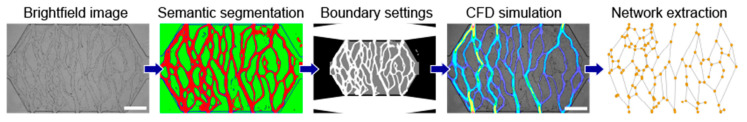
Processes of time-lapse images of spontaneously formed lumens. The original images were taken by brightfield microscopy. Semantic segmentation was used to distinguish lumens from nonlumens. The colors of the segmentation image show three classes (red: lumen, green: nonlumen, blue: lumen wall). To simulate flow based on the segmented lumens (binary images), an inlet/outlet channel geometry was added and meshed. The velocity field obtained from the CFD simulation was merged with the original image. Finally, network graphs were obtained from the segmented lumens, and the weights were calculated using the velocity field. Scale bar = 500 μm.

Before automatically segmenting all the brightfield images obtained from the experiments, manual semantic segmentation of three classes (lumen, matrix, and lumen wall) was performed on the 10 selected brightfield images obtained from the experiments to train the neural network. The authors empirically segmented by manually tracing the 10 chosen images in CLIP STUDIO Pro V2.0 (CELSYS, Tokyo, Japan) into RGB images as the ground truth data in which three color channels correspond to the three classes, as shown in Figure 2. These ground truth image data were transformed into 160 images/epochs of 224 × 224-pixel augmented images (randomly positioned, horizontally flipped and rotated by 90, 180, or 270 degrees) using MATLAB R2022b (or later) with the Deep Learning Toolkit (MathWorks, Natick, MA, USA). The augmented images were trained for 20 epochs on a net that replaced the input layer and 1st convolution layer of pre-trained DeepLab3+ based on MobileNet-V2 to adapt to monochrome images.

Using the above-trained network, semantic segmentation of the time-lapse images of the lumen was performed in MATLAB to obtain binary images representing the area occupied by the lumens. The change in lumen area was calculated from the sum of the ‘true’ pixels of these binary images. The obtained images were converted to a 2D mesh using im2mesh (https://www.mathworks.com/matlabcentral/fileexchange/71772, accessed on 29 March 2024), also in MATLAB. Then, FEATool V1.16 (Precise Simulation, Hong Kong, China) was used to set the boundary conditions and calculate the velocity field inside the lumen using a hydrodynamic simulation based on the Navier-Stokes equation. The wall shear stress (WSS) was calculated from the spatial velocity gradient in the normal vector direction of the lumen wall nodes.

### 2.5. Network Extraction and Analysis

The binary images of the lumen region obtained through semantic segmentation were skeletonized using standard functions of the MATLAB Image Processing Toolbox. Distance maps of the lumen images were also obtained. Next, the pixels of the skeletonized images were separated into the nodes and edges that make up the graph (network) using the same toolbox’s standard morphological operations on binary images. Then, the lumen region corresponding to each edge was reconstructed by inverse distance transformation of the distance map values masked by the edge pixels, representing the lumen’s half-width corresponding to the edge. The lumen region corresponding to each edge was used to mask the CFD result data value matrices and to extract the WSS and flow rate corresponding to the edge. The adjacency matrix was weighted with the WSS ratio $R_{\tau }$ or flow rate ratio $R_{Q}$:

(1) $$R_{\tau }=\sum_{i\in W}\tau_{i}\;÷\;\frac{8\mu U_{inlet}}{\frac{A_{lumen}}{d_{ch}}}$$

(2) $$R_{Q}=Q/(U_{inlet}w_{inlet})$$

where W is the index set of pixels of the lumen walls corresponding to the edge, $\tau_{i}$ is the WSS at the position of the i-th pixel, μ is the viscosity, $U_{inlet},w_{inlet}$ is the velocity and width of the inlet of the culture chamber, $A_{lumen}$ is the area of the whole lumen, $d_{ch}$ is the distance between the pillars defining two perfusion channels, and Q is the flow rate at the lumen corresponding to the edge. Finally, the adjacency matrix weighted with $R_{\tau }$ or $R_{Q}$ and the node coordinates were output to the MAT files.

The MAT file of the adjacency matrix and node coordinates was loaded in the R 4.3.3 software environment, and the *igraph* package V2.1.2 (https://CRAN.R-project.org/package=igraph, accessed on 29 March 2024) was used to construct the network diagram, weighted degrees (strengths), and betweenness centrality.

## 3. Results and Discussion

### 3.1. Microfluidic Cell Culture System Design

We performed medium circulation and CO_2_ incubation on-chip and combined them with a pillar-separated microfluidic 3D culture chamber. The system was compact, as shown in Figure 1c, and allowed long-term perfusion at constant flow rates and continuous observation under a conventional inverted microscope. Video S1 shows one example of the outcome of this system: a 1-h interval time-lapse recording of long (2 mm) lumens maintained for 44 days.

The first step in spontaneous lumen formation in vitro is vasculogenesis, i.e., forming a meshwork of single endothelial cells. Interstitial flow in the hydrogel is suitable for supporting vasculogenesis. However, active perfusion is required to maintain the lumen by providing the WSS required for physiological remodeling of the lumen walls, especially when the long lumens are developed and the interconnecting channels and pressure drops are rapidly reduced. As shown in Figure 1a, six Braille pins to the microfluidic device displaced the PDMS microchannel in peristaltic motion to perfuse the cell culture chamber. A braille cell is a replacement part that forms one braille character of a refreshable braille display, a text output device for visually impaired computer users. This makes braille cells compact and readily available, unlike other actuators mainly used in active microfluidic systems. In addition, the microfluidic channels used in this system require only a single layer, and the PMMA/PDMS components can be made using a molding process, making them suitable for mass production.

To achieve pulsation-free flow, two rows of the braille pumps are driven in parallel, with their phases shifted so that their dead periods do not overlap [34], and the meander flow resistor shown in Figure 1d is inserted into the microfluidic circuit. This allowed us to mimic blood flow in capillaries. The perfusion scheme can be switched by turning on/off the perfusion mode switching valve with a braille pin, as shown in Figure 1e. The microfluidic channel has a layout in which two perfusion channels sandwich the cell culture chamber in the middle. When the pin of the braille valve is open, and these two perfusion channels are connected in series, interstitial flow is generated between the two channels due to the pressure drop. On the other hand, when the valve is closed, the flow passes through the culture chamber. The interstitial flow through both channels until the lumen has a path that prevents excessive pressure from being applied to the endothelial cells, thereby inhibiting lumen formation. Many microfluidic 3D vascular culture systems have been proposed, but most use syringe pumps or passive fluid drives (hydrostatic or Laplace pressure), which are disadvantageous for long-term imaging. The former is complicated to handle under a microscope. It requires media replenishment and effluent disposal, while the latter is difficult to control regarding the direction of perfusion and flow rate.

To the authors’ knowledge, almost all conventional microfluidic 3D vascular cultures have a lumen length in the perfusion direction of less than 1 mm. A short lumen in the flow direction eliminates the need for active perfusion. At the same time, the experiment is likely to terminate when the entire flow path becomes a lumen within 1~3 weeks. We generated a lumen with a flow-directed length of 2 mm and reproduced the remodeling processes for more than four weeks, as described below. This success is likely because the active control of interstitial flow changes the shear stress applied to the endothelial cells and promotes sprouting and pruning by angiogenesis.

We also switched the direction of perfusion every 24 h. This method was effective in smoothing the gradient of mass transport and mechanical stimuli until the lumen matured by controlling the perfusion direction, allowing us to culture the vascular network in a larger culture chamber than in the previous studies.

### 3.2. Lumen Segmentation and Definition of the Stages of Long-Term Microvascular Perfusion Culture

After recording the spontaneous formation of the luminal network in vitro and its temporal evolution, the next step is to identify its morphology, obtain the morphological parameters, and classify the time-evolutional stages of the lumen network. In addition, the hydrodynamic properties of the luminal network should be analyzed to determine how the remodeling process optimizes them. Figure 2 shows an overview of the processing of the lumen images in this study: semantic segmentation of the lumens from time-lapse brightfield images, estimation of the segmentwise flow rate and WSS using image-based computational fluid dynamics (CFD), and extraction of network diagrams via morphological operations. Video S2 shows a montage of the semantic segmentation of the lumen, the velocity field in the lumen calculated using image-based CFD, and the extracted network for a 44-day lumen culture. In this example, 35 brightfield images from culture days 10~45 were automatically processed.

The in vitro spontaneous lumen reflects the network optimization process of the cell (population) depending on the flow inside the lumen or the properties of the gel matrix. Therefore, it cannot be predefined in shape, resulting in a lumen network that can take a variety of shapes. Extracting and quantifying features from these different shapes and evaluating their correlation with hydrodynamic parameters such as WSS are necessary to assess lumen shape optimization. On the other hand, conventional lumen detection and analysis methods are mainly based on the assumption of specific tissues (e.g., retina) in vivo. Imaging methods that include flow (e.g., tomography and Doppler imaging) are used in vivo. Nevertheless, their cost-effectiveness and throughput are too low to be applied to in vitro systems, and they do not take advantage of in vitro advantages. In addition, in vitro luminal characteristics often fall outside the performance range of conventional analytical methods. Therefore, it is necessary to establish unique in vitro analysis methods.

Fluorescence microscopy has been used for lumen imaging in previous reports on spontaneous lumen formation in vitro. Many reports use tracers (microspheres, molecular markers such as FITC-dextran) and fluorescently labeled cells (cell tracking dye stains, fluorescent protein-expressing HUVECs (FP-HUVECs)) as the fluorescence source. However, microspheres have the problem of remaining in the lumen, and FITC-dextran has the problem of blurring due to diffusion, although it provides good contrast for the luminal morphology. The cell tracking stains fade with proliferation, making long-term time-lapse recording difficult. The FP-HUVECs seem more effective in ensuring semantic segmentation, but their effectiveness was limited because all cells did not fluoresce uniformly. In addition, in our experiments, the long-term culture of FP-HUVECs did not result in lumen remodeling, suggesting that the tendency toward large passage numbers may be the cause.

Instead of using brightfield images without fluorescence, we used a deep neural network (DNN) to learn images of the lumen. We could automatically and quickly extract regions considered the lumen from hundreds of time-lapse images. However, it was necessary to pre-train the DNN with ground truth data while avoiding overfitting, a common problem with DNNs. We first used a small convolutional neural network (CNN; convolution layers = 3) to learn a tiny portion of the image (100 pixels per side) with a clear lumen boundary and then segmented another small image output by the CNN. The process was repeated by training with a revised CNN segmentation output of other tiny images. The size of the image and CNN were gradually increased during this repetition.

We also implemented an image processing pipeline in a MATLAB environment that combines DNN-based semantic segmentation with CFD processes (meshing and solving partial differential equations) and image morphology for network extraction. Traditionally, CFD has been performed in separate software for image segmentation and feature/network extraction. However, the intervention of different software tends to increase the processing time and requires unnecessary development to adapt to each input/output format. Therefore, the ability to use a consistent pipeline from image recognition to network extraction contributed significantly to the automatic batch processing of many images.

**Figure 3:**
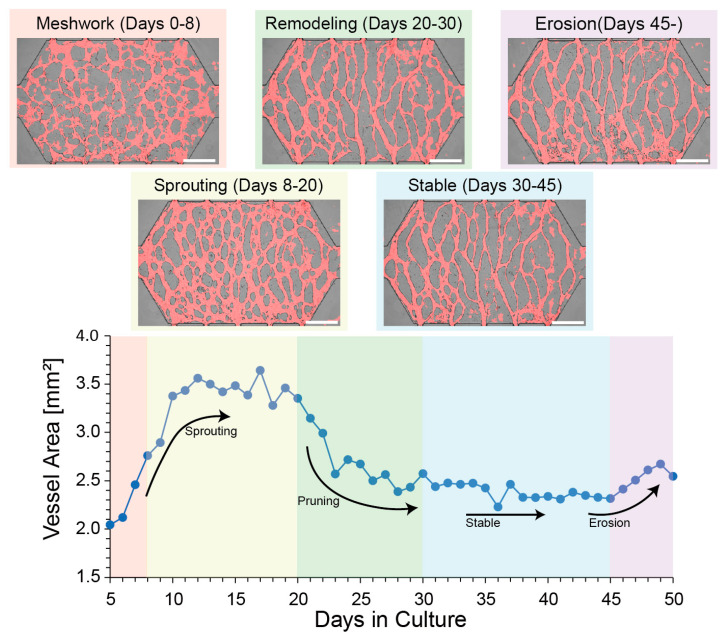
The morphological stages of HUVECs or the lumens they form during microfluidic perfusion culture. The temporal evolution of the luminal morphology can be qualitatively classified into the following stages: (1) Meshwork (Days 0~7, 8): cells aggregate and vasculogenesis occurs. A mesh-like isotropic lumen network emerges, and the lumen area increases. (2) Sprouting (Days 7, 8~20): with increased flow through the lumen, branching of the lumen is promoted, and anisotropy in the luminal network occurs. The total area of the lumen increases. (3) Remodeling (days 20~30): some lumens are trimmed, and the total area of the lumen begins to decrease. (4) Stable (days 30~45): the lumen wall does not move, and the cells remain viable and almost constant in shape. The total area of the lumen also remained unchanged; (5) Erosion (days 45~): HUVECs that grew monolayers in the perfusion channel migrated into the lumen and eroded the ECM outside the lumen. The total lumen area increases. Scale bar = 500 μm.

Figure 3 shows the relationship between the morphological stage of typical spontaneous lumens and the lumen area, an index directly obtained by lumen segmentation using a DNN. Almost all lumens successfully cultured in this system followed the process of meshwork → sprouting → remodeling → stable → erosion, although the duration of the process varied. In particular, the period from the end of pruning to the start of stabilization varied between 25 and 30 days, with a range of around 5 days, and the period when erosion begins also varied (See also Figure 5a). The HUVECs are dispersed and seeded in a fibrin–collagen gel and first form a reticular structure. The HUVECs initially distributed in fibrin–collagen gel first make their meshwork. By providing interstitial flow, they formed a lumen that penetrated the columns on both sides, and the lumen area increased during this stage. The area was further increased by flow through the lumen, which induced sprouting and development of a luminal network along the direction of perfusion. In remodeling, pruning became more dominant than sprouting, and the lumen area decreased. Then, the lumen movement decreased, i.e., the lumen became stable, but there was no cell death during this stage (experiments have shown that the lumen collapses when cells die).

The stages described thus far (from meshwork to remodeling) are considered to reproduce the physiological temporal development of lumens. However, the erosion stage does not. It is a phenomenon in which the HUVECs grow in a monolayer outside the hydrogel (perfusion channel shown in Figure 1d), migrate through the lumen, and erode the ECM. The invasion of cells causes the opposite phenomenon to occur during remodeling. The area of the lumen begins to increase again due to cell invasion. In other words, this phenomenon is the opposite of remodeling. This can be explained as a state in which the cells cultured in a cell culture vessel become overconfluent. This is not an optimization of the lumen network but a limitation unique to this system.

### 3.3. Evaluation of Network Optimization as Wall Shear Stress/Flow-Rate-Weighted Centralities

**Figure 4:**
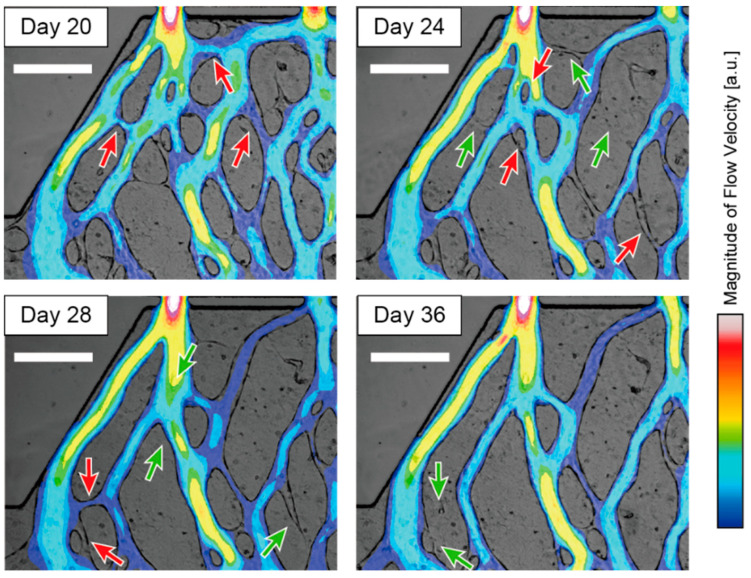
Example of pruning in the remodeling stage of an in vitro spontaneous lumen network. The brightfield image is overlaid with a velocity magnitude false color image. The same location in culture was photographed on days 20, 24, 28, and 36. The direction of perfusion is from top to bottom. The lumen indicated by the red arrows in the photograph has disappeared, as indicated by the green arrows in the next panel. Scale bar = 300 μm.

From the time-lapse images of the lumen structures, we observed that the remodeling processes depended on the flow conditions in the lumens. Figure 4 shows a typical remodeling of the lumen network, where redundant segments are pruned, and a similar trend was observed in other samples. By comparing the image of the lumen walls and the flow velocity field obtained from the CFD simulation, we can see the relationship between the flow and the segment to be pruned. The preferentially pruned lumen tends to be arranged to bridge the two thicker, higher-flowing lumens, and the lumen itself has a low flow rate. In terms of geometry, a lumen that is not oriented along a high-flow lumen and branched perpendicularly is more likely to be preferentially pruned because it is less likely to follow the streamline of the flow that is causing the high flow, as implied by a study that suggested the effect of differences in blood flow between neighboring vessels on the pruning process [35]. In addition, the easy pruning of lumens at angles close to the right of the mainstream may increase the flow directions anisotropy of the lumen network and improve the efficiency of the mass transport through the lumen network.

This phenomenon of pruning of lumens that are not oriented in the main perfusion direction has not been confirmed by microscopy images of other in vitro spontaneously forming lumens, such as those shown in the previous results [18,21,22,24]. This may be because the microfluidic system used to obtain the previous results was hydrostatically driven, and the incubation time was limited. In contrast, our system employs active perfusion to achieve long-term culture.

**Figure 5:**
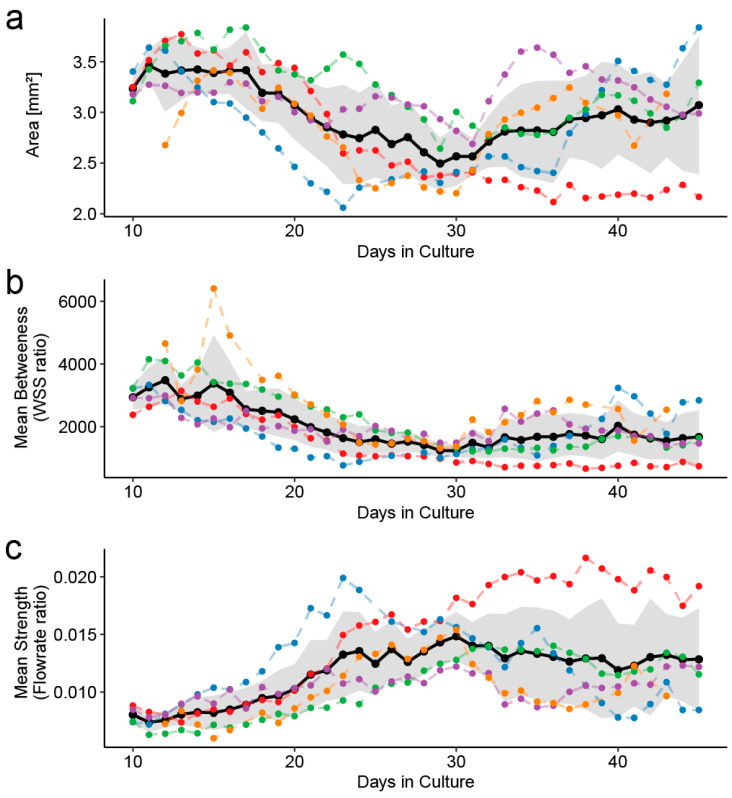
Time evolution of metrics of vascular remodeling computed from the time-lapse images. The colored dashed lines indicate the values for each sample, the black lines indicate the average values for all samples, and the bands indicate the 95% confidence intervals. (**a**) Total area of lumens. (**b**) Cost centralities of the network extracted from lumens: mean betweenness centrality of WSS ratio-weighted graphs. (**c**) Importance centralities of the network extracted from lumens: mean node strengths (i.e., degree of weighted network) of the WSS-weighted network.

We also evaluated the lumen network’s effectiveness in terms of measures of the network diagram’s centrality extracted from the time-lapse images and weights obtained from the CFD simulation results. Figure 5 shows the temporal evolution of the total lumen area and the average of the two network node centrality types.

As mentioned earlier, the total lumen area shown in Figure 5a indicates that lumen sprouting or remodeling occurred. However, it does not indicate how the lumen shape relates to the wall shear stress (WSS) distribution and flow rate distribution. On the other hand, Figure 5b shows the average value of the network’s betweenness centrality with the weight of the WSS ratio, which is the integral value of the WSS magnitude along the lumen wall divided by the reference WSS value calculated from the total lumen area. The mean value of the betweenness centrality is significantly lower in the remodeling stage.

Here, the definition of betweenness centrality of a node is <the sum of the weights along the shortest path between all node pairs via that node>, divided by <the sum of the weights along the shortest path between all node pairs (without via node)>. This value indicates how much the node is the bottleneck of the entire network due to the WSS, a cost factor of material transport energy. In other words, as shown in Figure 5b, a decrease in the average betweenness centrality means that the influence of the WSS decreases in the network. Microscopically, this result suggested the disappearance of lumens that increase cost rather than flow by branching out from lumens with high flow (therefore, high WSS). Since such lumen loss behavior results in a more uniform flow distribution, the results in Figure 5b are at least consistent with reports that the zebrafish microvascular network is optimized to have a uniform overall flow distribution [28].

In addition, Figure 5c shows the average value of strength centrality in the network using the flow rate ratio as the weight. The flow rate ratio is the flow rate through a lumen segment divided by the total flow in the lumen network. A node’s strength centrality is the sum of the weights of all edges directly connected to that node. Unlike mean betweenness, the mean strength centrality is a statistical value of importance centrality and quantifies the degree to which “important” nodes occupy the perfusion. In other words, it is considered a valid indicator of the overall optimization of flow efficiency. As shown in Figure 5c, the strength centrality increases during the remodeling process. This aligns with the belief that the lumen network is optimized to increase mass transport efficiency (cf. [13], for example). In addition, compared with pure morphological network optimization metrics such as architecture characteristics (vessel number, size, arrangement, and diameter) [36], fractal and power-spectrum analysis [37], and self-similarity analysis and branching properties [38], the centralities of flow parameter-weighted graphs are metrics that directly correlate the morphological characteristics of the lumens with the degree of hydrodynamic stimulation. Ultimately, Figure 5 shows that the network analysis of the lumen network demonstrates that both the transport efficiency and cost uniformity of the lumen network are improved through remodeling.

## 4. Conclusions

We developed an in vitro model of long-term microvascular remodeling processes with a compact integrated microfluidic system. Adaptive interstitial flow and feedthrough perfusion switching in one microfluidic system was the key to a successful culture of spontaneously formed, naturally branched lumens for over one month. We obtained many large-area (2 mm × 3 mm) long-term (more than 30 days, with a maximum of 150 days per run) time-lapse image datasets of the in vitro luminal network using this microfluidic system. These datasets showed in vivo-like, physiologically reasonable (low energy dissipation, flow uniformity) lumens and recorded the self-assembly process of vascular endothelial cells. This topic is expected to be the subject of future analyses other than those presented in this paper.

We conducted these long-term culture experiments in a typical cell culture laboratory, implying that the microfluidic system is highly adaptable to experimental environments with many limitations, such as space laboratories.

We have also developed an automatic image analysis pipeline, including semantic segmentation, CFD, and weighted graph extraction. Based on the cost- and importance-weighted network graphs extracted from the microscope images of lumens, we evaluated the flow velocity uniformity, WSS, and mass transfer efficiency. The optimization of the remodeling process can be explained by the decrease in the betweenness centrality of the WSS-weighted network and the increase in the strength centrality of the flow-rate-weighted network. These results indicate the possibility of explaining the process of lumen formation from the viewpoint of network optimization, which is a practical approach to understanding the organism as a theoretical system. The model constructed in this study represents the function and dysfunction of endothelial function, and further network analysis of the model will lead to elucidating the mechanism of endothelial dysfunction.

The lumen model created in this study can also be interpreted as part of an organ-on-a-chip. From a practical point of view, if the results of this study can be applied to create lumens with pathologically and hydrodynamically distinctive morphologies, such as aneurysms, stenosis, stroke, and atherosclerosis, the reliability of organ-on-a-chip-based drug testing can be improved.

## Data Availability

The original contributions presented in the study are included in the article/Supplementary Material, further inquiries can be directed to the corresponding author.

## Supplementary Materials

The following supporting information can be downloaded at: https://www.mdpi.com/article/10.3390/mi16010014/s1, File S1: MATLAB Script; Video S1: Time-lapse recording of lumens maintained for 44 days. Video S2: Montage of the semantic segmentation of the lumen, the velocity field in the lumen calculated using image-based CFD, and the extracted network for a 44-day lumen culture.
