# Supplementary material for: In Vitro Model of Vascular Remodeling Under Microfluidic Perfusion
Source: Micromachines (Basel). 2024 Dec 26;16(1):14. doi: 10.3390/mi16010014 (PMC11767722; doi:10.3390/mi16010014)
Supplement: Supplementary file 1 [file micromachines-16-00014-s001.zip › File S1.pdf]

Supporting Information

In Vitro Model of Vascular Remodeling Under Microfluidic Perfusion

Kotaro Nishikata, Kimisato Doi, Nobuyoshi Kaneoya, Masataka Nakamura and Nobuyuki Futai *

Department of Mechanical Engineering, Shibaura Institute of Technology, 3-7-5 Toyosu, Koto-ku, Tokyo 135-8548, Japan; nb22110@shibaura-it.ac.jp (K.N.); aa20055@shibaura-it.ac.jp (K.D.); md23038@shibaura-it.ac.jp (N.K.)

* Correspondence: futai@shibaura-it.ac.jp; Tel.: +81-3-5859-8016

SI. 1. MATLAB Script

The MATLAB script used for semantic segmentation, network extraction, and analysis is shown below.

Semantic segmentation

```
CNNtest0Common

load("netDLV.mat", "netDLV");
infile = testdir + "¥¥" + filenameTestSeqImage + fileSeqExt;
outfile = testoutdir + "¥¥" + filenameTestSeqImage;

nPage = length(imfinfo(infile));
for k = 1:nPage
    I = imread(infile,k);

    C = semanticseg(I, netDLV);
    OV = labeloverlay(I, C);
    %imwrite(OV,sprintf(outfile + "%03d_ovl.png", k));

    if k == 1
        imwrite(OV, outfile + ".tif");
    else
        imwrite(OV, outfile + ".tif", 'writemode', 'append');
    end

    RGB = class2RGB(C, classes);
    imwrite(RGB,sprintf(outfile + "%03d_mask.png", k));
end


function RGB = class2RGB(C, classes)
    R = class2BW(C, classes(1));
    G = class2BW(C, classes(2));
    B = class2BW(C, classes(3));
    RGB =cat(3, R, G, B);

    function BW = class2BW(C, cls)
        BW = uint8(C == cls);
        BW(BW>0) = 255;
    end
end
```

Network analysis for strength centrality

```
in_file_1 = "test_out_xxxx";
in_file_2 = "test_skel_xxxx";
filelist1 = dir(fullfile(in_file_1,'*.png'));
filelist2 = dir(fullfile(in_file_2,'*.png'));
nImages = length(filelist2);

cellG = cell(nImages,1);
cellX = cell(nImages,1);
cellY = cell(nImages,1);
cellAdj = cell(nImages,1);

mu = 1e-3;
chlen = 2e-3;
u_mean = 10e-6;
wid_inlet = 0.5e-3;
Q_inlet = u_mean * wid_inlet;
scale_im = chlen / 1200;

for i = 1:nImages
    bwName = filelist1(i).name;
    im_bw = imread(fullfile(in_file_1,bwName));
    skelName = filelist2(i).name;
    im_skel = imread(fullfile(in_file_2,skelName));
    vel = velo{i,1};
    Dist = bwdist_old(~im_bw);
    [madj, X, Y] = MakeGraph(im_skel, Dist, vel, scale_im, Q_inlet);
    madj(isnan(madj)) = 0;
    G = graph(madj);
    cellG(i) = {G};
    cellX(i) = {X};
    cellY(i) = {Y};
    cellAdj(i) = {madj};
end

plot(G, 'XData', X, 'YData', Y, 'EdgeLabel', arrayfun(@(x) sprintf("%.1g",
x), G.Edges.Weight));

save cellg.mat cellG
save cellx.mat cellX
save celly.mat cellY
save a.mat cellAdj

function [madj, X, Y] = MakeGraph(im_skel, Dist, velo, scale_im, Q_inlet)
    SE = strel("diamond",1);
    SE2 = strel("diamond",2);
    im_skel2 = bwmorph(im_skel,"spur");
    im_br = bwmorph(im_skel2,"branchpoints");
    im_en = bwmorph(im_skel2,"endpoints");
    im_nodes = im_br | im_en;
    im_node_dilated = imdilate(im_nodes, SE);
    im_seg = im_skel2;
    im_seg(im_node_dilated ~= 0) = 0;
    im_seg_label = bwlabel(im_seg);
    nSegments = max(im_seg_label,[],"all");
    im_node_label = bwlabel(im_node_dilated);
```

```
    nNodes = max(im_node_label,[],"all");
    s = regionprops(im_node_dilated, 'centroid');
    ctrds = cat(1, s.Centroid);
    X = ctrds(:, 1);
    Y = ctrds(:, 2);
    madj = zeros(nNodes);

    for i = 1:nSegments
        im_seg_i = (im_seg_label == i);
        im_seg_i_dil = imInvDist(im_seg_i, Dist);
        im_seg_i_velo = velo(im_seg_i_dil == true);
        [~,~,wids] = find(Dist.*im_seg_i);
        nSeg_wid_i = 2 * mean(wids);
        veloMean = mean(im_seg_i_velo(:));
        Q = veloMean * nSeg_wid_i * scale_im;
        im_seg_dilated = imdilate(im_seg_i, SE2);
        im_ep_i = im_node_dilated & im_seg_dilated;
        [~,~,v_nodeidx_i] = find(im_node_label(im_ep_i ~= 0));
        v_nodeidx_i = unique(v_nodeidx_i);
        if (length(v_nodeidx_i) >= 2)
            madj(v_nodeidx_i(1),v_nodeidx_i(2)) = Q/Q_inlet;
            madj(v_nodeidx_i(2),v_nodeidx_i(1)) = Q/Q_inlet;
        end
    end
end

function bw_dilated = imInvDist(bw_seg, im_dist)
    [r_bw, c_bw] = find(bw_seg);
    pixs_im = diag(im_dist(r_bw, c_bw));
    im2o = zeros(size(bw_seg), 'uint8');
    im2 = insertShape(im2o,"filled-circle",[c_bw r_bw pixs_im],
ShapeColor=[1 0 0], Opacity=1, smoothEdges=false);
    bw_dilated = logical(im2(:,:,1));
end
```

Network analysis for betweenness centrality

```
in_file_1 = "test_out_xxxx";
in_file_2 = "test_skel_xxxx";
filelist1 = dir(fullfile(in_file_1,'*.png'));
filelist2 = dir(fullfile(in_file_2,'*.png'));
nImages = length(filelist2);

cellG = cell(nImages,1);
cellX = cell(nImages,1);
cellY = cell(nImages,1);
cellAdj = cell(nImages,1);

mu = 1e-3;
chlen = 2e-3;
u_mean = 10e-6;

scale_im = chlen / 1200;

for i = 1:nImages
    bwName = filelist1(i).name;
    im_bw = imread(fullfile(in_file_1,bwName));
    wss_field = flipud(WSS{i,1});
```

```
    wssratio = wss_field / (8 * mu * u_mean * chlen / (sum(im_bw,'all') *
scale_im^2));
    Dist = bwdist_old(~im_bw);
    skelName = filelist2(i).name;
    im_skel = imread(fullfile(in_file_2,skelName));
    [madj, X, Y] = MakeGraph(im_skel, Dist, wssratio);
    G = graph(madj);
    cellG(i) = {G};
    cellX(i) = {X};
    cellY(i) = {Y};
    cellAdj(i) = {madj};
end

i = nImages;
plot(cellG{i}, 'XData', cellX{i}, 'YData', cellY{i}, 'EdgeLabel',
cellG{i}.Edges.Weight);

save cellg.mat cellG
save cellx.mat cellX
save celly.mat cellY
save a.mat cellAdj

function [madj, X, Y] = MakeGraph(im_skel,Dist,wss)
    SE = strel("diamond",1);
    SE2 = strel("diamond",2);

    im_skel2 = bwmorph(im_skel,"spur");
    im_br = bwmorph(im_skel2,"branchpoints");
    im_en = bwmorph(im_skel2,"endpoints");
    im_nodes = im_br | im_en;
    im_node_dilated = imdilate(im_nodes, SE);

    im_seg = im_skel2;
    im_seg(im_node_dilated ~= 0) = 0;

    im_seg_label = bwlabel(im_seg);
    nSegments = max(im_seg_label,[],"all");
    im_node_label = bwlabel(im_node_dilated);
    nNodes = max(im_node_label,[],"all");

    s = regionprops(im_node_dilated, 'centroid');
    ctrds = cat(1, s.Centroid);
    X = ctrds(:, 1);
    Y = ctrds(:, 2);

    madj = zeros(nNodes);

    for i = 1:nSegments
        im_seg_i = (im_seg_label == i);
        im_seg_i_dil = imInvDist(im_seg_i, Dist);
        im_seg_i_wss = wss(im_seg_i_dil == true);
        wssSum = max(1e-20, sum(im_seg_i_wss,"all"));

        im_seg_dilated = imdilate(im_seg_i, SE2);
        im_ep_i = im_node_dilated & im_seg_dilated;

        [~,~,v_nodeidx_i] = find(im_node_label(im_ep_i ~= 0));
        v_nodeidx_i = unique(v_nodeidx_i);
```

```
        if (length(v_nodeidx_i) >= 2)
            madj(v_nodeidx_i(1),v_nodeidx_i(2)) = wssSum;
            madj(v_nodeidx_i(2),v_nodeidx_i(1)) = wssSum;
        end
    end
end

function bw_dilated = imInvDist(bw_seg, im_dist)
    [r_bw, c_bw] = find(bw_seg);
    pixs_im = diag(im_dist(r_bw, c_bw));
    im2o = zeros(size(bw_seg), 'uint8');
    im2 = insertShape(im2o,"filled-circle",[c_bw r_bw pixs_im],
ShapeColor=[1 0 0], Opacity=1, smoothEdges=false);
    bw_dilated = logical(im2(:,:,1));
end
```
